# Melatonin Protects Intervertebral Disc from Degeneration by Improving Cell Survival and Function via Activation of the ERK1/2 Signaling Pathway

**DOI:** 10.1155/2019/5120275

**Published:** 2019-12-02

**Authors:** Jun Ge, Quan Zhou, Junjie Niu, Yingjie Wang, Qi Yan, Cenhao Wu, Jiale Qian, Huilin Yang, Jun Zou

**Affiliations:** ^1^Department of Orthopaedic Surgery, The First Affiliated Hospital of Soochow University, Suzhou, Jiangsu 215006, China; ^2^Department of Orthopedics Surgery, The Affiliated Huai'an Hospital of Xuzhou Medical University, Huai'an, Jiangsu 223002, China

## Abstract

Melatonin, a neuroendocrine hormone secreted by the pineal body, has a positive effect on intervertebral disc degeneration. The present study is aimed at investigating the biological role of melatonin in intervertebral disc degeneration and its underlying mechanism. A human nucleus pulposus cell (NPC) line was exposed to melatonin at different concentrations. Cell proliferation was measured by CCK-8 assay. Cell cycle and apoptosis were analyzed by flow cytometry. Western blot was performed to measure the protein expression of indicated genes. A rabbit model of intervertebral disc degeneration was established to detect the role and mechanism of melatonin on intervertebral disc degeneration. Our study showed that melatonin promoted NPC viability and inhibited cell arrest. Furthermore, melatonin treatment led to the upregulation of collagen II and aggrecan and downregulation of collagen X. Moreover, melatonin significantly elevated the activity of the ERK signaling pathway. Inhibition of the ERK1/2 signals reversed the role of melatonin in the regulation of NPCs both *in vitro* and *in vivo*. Melatonin increased NPC viability through inhibition of cell cycle arrest and apoptosis. Moreover, melatonin promoted the secretion of functional factors influencing the nucleus pulposus cell physiology and retarded cell degeneration. Our results suggest that melatonin activated the ERK1/2 signaling pathway, thereby affecting the biological properties of the intervertebral disc degeneration.

## 1. Introduction

It is predicted that up to 80% of adults in the United States would experience some form of low back pain over their lifetime, with approximately 5% of sufferers becoming chronically disabled [1]. Intervertebral disc degeneration (IDD) is considered a chief cause of low back pain [2–4].

Melatonin is a neuroendocrine hormone secreted by the pineal body and has a role in the regulation of circadian and seasonal rhythms [5]. In addition, melatonin is closely involved in the functional regulation of neuroendocrine, reproductive, immune, and cardiovascular systems [6–9]. Previous studies showed that melatonin promotes the proliferation and differentiation of osteoblasts and bone formation in the skeletal system. In contrast, melatonin inhibits osteoclast activity and reduces bone resorption. Moreover, melatonin promotes the differentiation of bone marrow mesenchymal stem cells (BMSCs) to osteoblasts. All these biological effects of melatonin are regulated by the melatonin receptor [4, 10–12]. In addition, the expression of MTl and MT2 melatonin receptors was observed in chondrocytes and BMSCs, suggesting that melatonin may regulate biological processes in these cells [13, 14].

Recently, studies on melatonin and chondrocytes have shown a closer relationship of melatonin with the skeletal system. Porcine articular chondrocytes were evaluated *in vitro* by Pei et al. in a pellet culture system. Their results showed that the melatonin treatment yielded chondrocyte pellets with a higher expression of chondrogenic markers both at the mRNA and protein levels. A hypertrophic marker remained low, suggesting that melatonin promoted the chondrogenic differentiation and reduced the osteogenic differentiation [15]. Lim et al. examined the effects of melatonin in hydrogen peroxide- (H_2_O_2_-) stimulated human chondrocytes. Melatonin markedly inhibited H_2_O_2_-stimulated cytotoxicity, iNOS, and COX-2 protein and mRNA expression as well as their downstream products, NO and PGE. Incubation of cells with melatonin decreased H_2_O_2_-induced Sirtuin 1 (SIRT1) expression. SIRT1 inhibition by sirtinol or SIRT1 siRNA reversed the effects of melatonin on proinflammatory cytokines and the expression of iNOS and COX-2. In rabbits with osteoarthritis, intra-articular injection of melatonin significantly reduced cartilage degradation, which was reversed by sirtinol. This study shows that melatonin exerts cytoprotective and anti-inflammatory effects in an oxidative stress-stimulated chondrocyte model and rabbit osteoarthritis model [16].

Nucleus pulposus cells (NPCs) of the intervertebral discs are chondrocyte-like cells, homologous to the chondrocytes in term of gene expression and function. In 2006, Turgut et al. [17] performed a pinealectomy on chickens, which accelerated the IDD process. Further studies [18–21] proved the positive and protective role of melatonin in the degenerative nucleus pulposus cells. Despite these encouraging reports, none of them has comprehensively demonstrated the underlying mechanisms by which melatonin triggers these positive effects of the melatonin on IDD both *in vivo and in vitro*, whether melatonin can be widely applied to patients with IDD or whether it has limitations and can only be applied to a few patients. The insufficient understanding on the specific therapeutic mechanisms of melatonin is bound to hinder the development of clinical applications of melatonin and the development of molecular biological treatment of IDD.

The current study is aimed at investigating the effect of melatonin on NPCs from the perspective of both cell survival and cell function and elucidating the underlying mechanism, in order to further promote the development of IDD molecular therapy.

## 2. Materials and Methods

### 2.1. Cell Culture

The human nucleus pulposus cell (NPC) line was purchased from ScienCell (Carlsbad, CA, USA, Cat. No.4800). Cell culture was performed as described in the protocol in our previous study [22]. Cells preserved in liquid nitrogen were quickly thawed in a preheated water bath (37°C) for 2 min. Then, the cells were washed in 5 mL of culture medium in a centrifuge tube. The supernatant was removed and cells were cultured in an incubator after centrifugation. When cells reached 90% confluency, they were passaged at a ratio of 1 : 2.

### 2.2. Determination of Cell Viability

Cell viability of NPCs was determined at 24 h and 96 h after the induction with 0.1, 0.5, 1, 5, 10, 50, and 100 *μ*M melatonin. The measurement procedure was as described in our previous study [22]: 10 *μ*L of Cell Counting Kit-8 (CCK-8) solution (Dojindo, Tokyo, Japan) was added to each well and the 96-well plates were incubated for 1 h at 37°C and 5% CO_2_. After that, the supernatants were carefully aspirated. To further lyse the cells and completely dissolve the precipitates, dimethyl sulfoxide (DMSO) was added to each well and the plates were shaken for 15 min. The absorbance was then measured at 450 nm. The background absorbance of the medium in the absence of cells was subtracted. The experiments were independently repeated at least three times.

### 2.3. Analysis of Cell Cycle

Propidium iodide (PI) staining was used to analyze cell cycle distribution as described in our previous study [22]. NPCs were digested by trypsin and centrifuged at 3000 rpm for 5 min. Then, cells were washed in ice-cold PBS and then centrifuged. The supernatant was carefully removed and cells were fixed in 70% ice-cold ethanol at 4°C overnight. Cells were collected and digested in RNase (50 ng/mL). PI (20 ng/mL) was added and cells were incubated at 37°C for 1 h. Finally, the cell cycle was analyzed by flow cytometry.

### 2.4. Analysis of Apoptotic Cells

A double-staining assay was performed using an Annexin V-FITC/PI kit to evaluate the percentage of apoptotic cells. NPCs were treated with different concentrations of melatonin for 24 h before harvest. We follow the protocol in our previous study [22]. The cells were washed with 4°C PBS prior to being resuspended in a total volume of 500 *μ*L binding buffer. Then, 5 *μ*L of Annexin V-FITC solution and 5 *μ*L of PI were added to each sample. The cell suspension was kept on ice in the dark for 5 min. After that, the analysis was carried out by flow cytometry. Relative apoptosis rate was compared with the control group.

### 2.5. Western Blot Analysis

The cells were washed three times with PBS and suspended in ice-cold lysis buffer (Bio-Rad, Hercules, CA, USA). The lysates were separated by 12% SDS-PAGE, and the proteins of equal quantity were transferred to nitrocellulose membranes. Membranes were blocked with Tris-buffered saline with Tween-20 buffer (Thermo Fisher Scientific, Waltham, MA, USA) containing 5% nonfat milk (Yili, China) and incubated overnight at 4°C with collagen II, collagen X (Abcam, Cambridge, MA, USA), aggrecan, Bcl-2, Bax, caspase-3 (Santa Cruz, Dallas, TX, USA), p21, p27, CDK2, CDK4 (Cell Signaling Technology, Danvers, MA, USA), p-ERK1/2, and ERK1/2 (Abcam, Cambridge, MA, USA) primary antibodies. The following day, the membranes were washed and incubated with the corresponding secondary antibodies at room temperature (22-28°C) for 1 h. An enhanced chemiluminescence detection system (Thermo Scientific, MA, USA) was finally used to determine the emission of the membrane. The experiments were performed in three independent replicates. The GAPDH expression level was used as an internal control. The Western blot results were quantified with an image analyzer (Quantity One-4,2,0, Bio-Rad, Hercules, CA) and were normalized to GAPDH immunostaining. The relative protein expression was compared with the control group.

### 2.6. Establishment of a Rabbit Intervertebral Disc Degeneration Model

One-year-old New Zealand white rabbits, weighing 3.0–3.2 kg, were selected (*n* = 15). The rabbits were anesthetized by intramuscular injection of ketamine and xilazine and placed in a lateral decubitus position. Aseptic technique was applied for all surgical procedures. Fifteen-centimeter hair over the surgical field was shaved. Through a left retroperitoneal approach, the third lumbar processus transversus (L3) was exposed and removed from the roots, resulting in the exposure of the L3/4 intervertebral disc. This disc was punctured by a 16-gauge needle to a depth of 5 mm from the anterolateral fiber ring for 5 seconds. The needle was then pulled out without disturbing the spinal cord. Gentamicin (80,000 U) was used before and after the surgery. After the surgery, all the rabbits were allowed to move freely in a cage. Magnetic resonance imaging (MRI) was used four weeks after surgery to verify the establishment of the IVD model (0 week). After verifying the degeneration, the rabbits were randomly divided into 3 groups, including the melatonin group, the melatonin+ERK inhibitor group, and the saline group. Rabbits were conventionally exposed and their intervertebral discs were injected with 20 *μ*L saline, melatonin (2 mg/mL), and melatonin plus U0126 (0.4 mg/kg) (Sigma, St. Louis, MO, USA), into the L3/4 intervertebral disc, respectively. Then, the surgical wounds were sutured. After the injection, all the rabbits were allowed to move freely in a cage. All the experimental procedures were performed at the Laboratory Animal Center of Soochow University. All the experimental procedures were approved by the Ethics Committee of the First Affiliated Hospital of Soochow University and were carried out in strict accordance with the Declaration of Helsinki (1964) and the Laboratory Animal Guidelines for Ethical Review of Animal Welfare (GB/T35892−2018, China).

### 2.7. MRI Examination

MRI was performed to assess the degree of intervertebral disc degeneration at four and eight weeks after injection. Sagittal T2-weighted images were obtained using a 1.5 T MRI (GE HDx, Milwaukee, WI, USA). In the T2-weighted images, 5 discs (5 animals) per group were classified according to the modified Thompson classification from grades I to IV (I, normal; II, minimal decrease of signal intensity but obvious narrowing of high signal area; III, moderate decrease of signal intensity; and IV, severe decrease of signal intensity) [10].

### 2.8. Histological Examination

Rabbits were sacrificed after an eight-week MRI examination. The L3/4 intervertebral discs were harvested, fixed in 10% neutral formaldehyde, and decalcified in 10% EDTA for 4 weeks. The tissues were then horizontally cut into five slices, 4 *μ*m in width. After hematoxylin and eosin (H&E) staining, the slices were observed under a microscope. For immunohistochemistry detection, sections were incubated with primary antibodies specific to ERK (EterLife, UK), collagen II (Novus Biologicals, CO, USA), and collagen X (Biosynthesis Biotech, China). The obtained images were captured on an image analysis system and analyzed by Image Pro Plus 6.0 software (Media Cybernetics, Baltimore, MD, USA). Integrated optical density was calibrated, and the area of interest was established. The mean optical density was defined as the integrated optical density divided by area.

### 2.9. Statistical Analysis

All quantitative data are presented as mean ± S.D. For parametric data, statistical analyses were performed by one-way ANOVA. For nonparametric data, the Kruskal-Wallis test was performed. Differences with values of *p* < 0.05 were considered statistically significant.

## 3. Results

### 3.1. Melatonin Enhances NPC Viability

**Figure 1:**
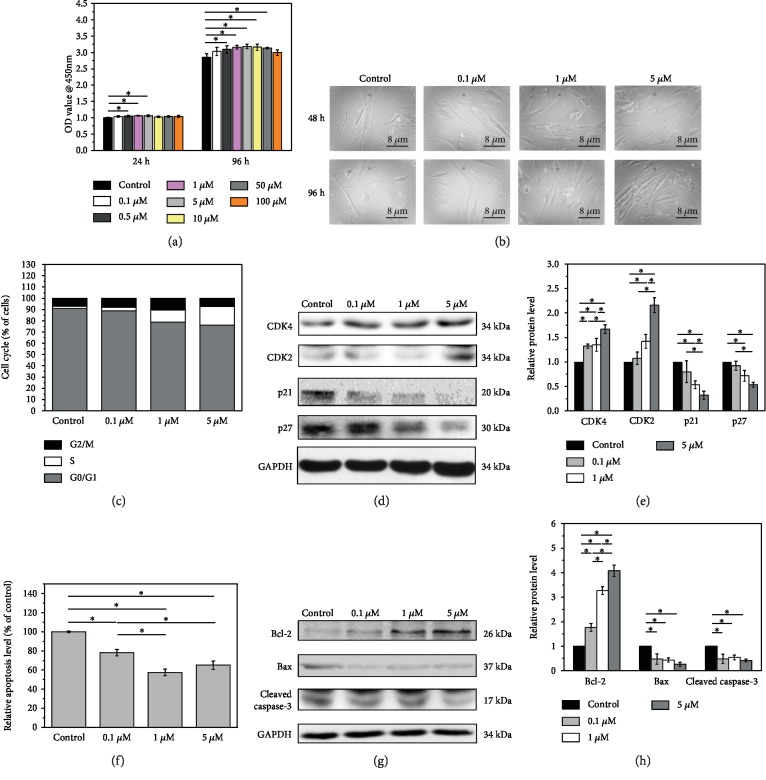
(a) Melatonin enhanced NPC viability. Cell viability of each group with 0.1, 0.5, 1, 5, 10, 50, and 100 *μ*M melatonin at both 24 h and 96 h. Significant viability was observed in cells with melatonin treatment than that in untreated ones in both 24 h and 96 h (^∗^*p* < 0.05 compared to the control group). (b) Melatonin alleviated the morphological changes in a dose-dependent manner. The cells were short and round in the control group. With increasing concentration, the changes to cell morphology were more obvious. Cells became bigger and long spindle shape. (c) Melatonin promoted the cell cycle progression. Melatonin-treated cells showed an increased percentage of cells in the S phase and decreased G0/G1 populations, a promoting of cell cycle progression in a dose dependent manner. (d) Western blot showed that the expression of the cell cycle-related proteins, CDK2 and CDK4, was increased, while p21 and p27 were decreased. (e) Quantified analysis of cell cycle-related protein Western blot result (^∗^*p* < 0.05). (f) Melatonin decreased the apoptosis of NPCs. A noticeable decrease of NPC apoptosis was observed, which was significantly promoted by cotreatment with 0.1, 1, and 5 *μ*M melatonin (^∗^*p* < 0.05). (g) Western blot showed that melatonin significantly ameliorated the expressions of apoptosis-related proteins, Bax and caspase-3, and it also induced the expression of Bcl-2. (h) Quantified analysis of apoptosis-related protein Western blot result (^∗^*p* < 0.05).

Initially, to investigate the effect of melatonin on nucleus pulposus cell behavior, we treated the cells with 0.1, 0.5, 1, 5, 10, 50, and 100 *μ*M melatonin for 24 h and 96 h. NPC viability was analyzed using the CCK-8 assay. The viability of NPCs showed slight but significant increases at a concentration of melatonin between 0.5 *μ*M and 5 *μ*M compared with the control group both in 24 h and 96 h (Figure 1(a), *p* < 0.05). In 96 h, slight but significant increases were also detected with a concentration between 5 *μ*M and 50 *μ*M (Figure 1(a), *p* < 0.05).

### 3.2. Melatonin Improves NPC Morphology

On the basis of these results, NPCs treated with melatonin at 0.1, 1, and 5 *μ*M were chosen for further study. Changes in their morphology were observed under a microscope. Compared with the control group, the NPC morphological changes could be observed in a dose-dependent manner. The cells changed from short and round to long spindle or polygonal with the treatment of melatonin (Figure 1(b)).

### 3.3. Melatonin Reduces NPC Cell Cycle Arrest and Apoptosis and Improves NPC Survival

The effect of melatonin on NPC cycle progression was analyzed using flow cytometric analysis. Cell cycle analysis revealed that melatonin at <5 *μ*M dose-dependently increased the percentage of cells in the S-phase and decreased the G0/G1 populations compared with the control group, suggesting that melatonin affected the G0/G1 to S phase transition (Figure 1(c)). These data suggested that melatonin could promote cell cycle progression, which was consistent with the CCK-8 assay results. We next investigated the cell cycle-related protein levels in cells treated with various concentrations of melatonin using Western blot. The expression of the CDK2 and CDK4 proteins was increased, while the cyclin-dependent kinase inhibitors p21 and p27 were decreased in a dose-dependent manner (Figure 1(d)). Quantified analysis showed that melatonin at 1 *μ*M and 5 *μ*M showed a significant effect on the expression of the cell cycle-related proteins (Figure 1(e), *p* < 0.05), while a slight but not significant increase of p21 at a low concentration of melatonin (0.1 *μ*M) was observed (Figure 1(e), *p* > 0.05). These results were consistent with the G0/G1 population in the flow cytometric analysis.

In addition, we examined the role of melatonin in nucleus pulposus cell apoptosis and found that melatonin decreased the apoptosis of NPCs in a dose-dependent manner. Compared with the control group, a noticeable decrease of cell apoptosis was observed in the NPCs, which was significantly increased by cotreatment with 0.1, 1, and 5 *μ*M melatonin (*p* < 0.05, Figure 1(f)). We also examined the apoptosis-related proteins by Western blot. In comparison to the control group, melatonin decreased the expressions of Bax and active caspase-3 proteins greatly; furthermore, melatonin had higher levels of the expression of Bcl-2 protein. The Western blot and the corresponding quantified analysis further demonstrated that melatonin could significantly inhibit the apoptosis of NPCs to enhance their activity (Figures 1(g) and 1(h), *p* < 0.05).

### 3.4. Melatonin Protects NPCs from Degeneration

**Figure 2:**
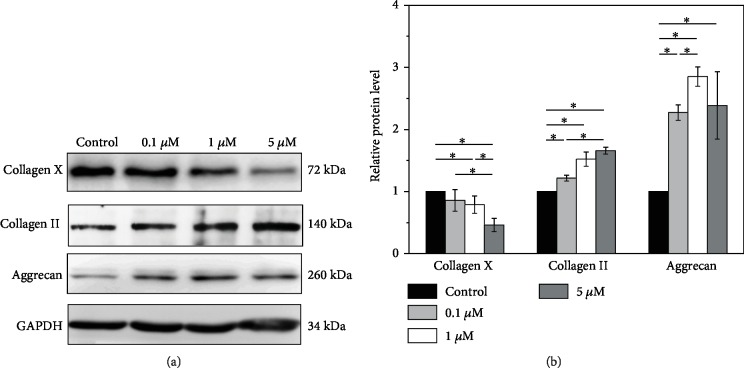
(a) Effect of melatonin to determine the expression levels of matrix proteins of NPCs using Western blot. The expression of collagen II and aggrecan proteins is increased and collagen X is decreased in NPCs when compared to the control. (b) Quantified analysis of Western blot result (^∗^*p* < 0.05).

Furthermore, to determine the expression levels of matrix proteins of NPCs, Western blot analysis was applied. Collagen and aggrecan are the two major extracellular matrix components of intervertebral discs and have been shown to play critical roles in normal disc function. We found that melatonin dose-dependently increased the expression of collagen II and aggrecan and decreased that of collagen X (Figures 2(a) and 2(b), *p* < 0.05). Thus, melatonin alleviated the degeneration of NPCs through modulation of the related proteins.

### 3.5. Melatonin Exerts Its Function via the ERK Pathway

**Figure 3:**
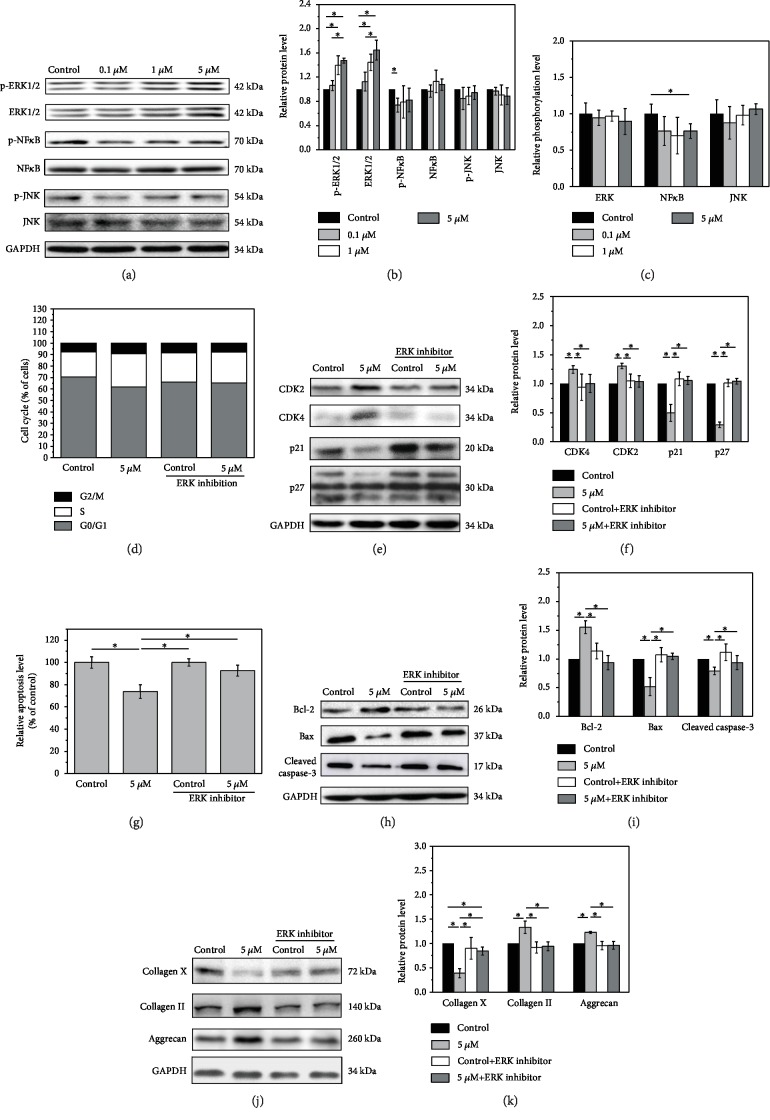
Melatonin, via the ERK pathway, regulates NPCs. (a–c) Melatonin significantly increased ERK1/2 activity above the concentration of 1 *μ*m. A slight inhibition of phosphorylated NF-*κ*B protein expression was detected at 1 *μ*M. A decrease in phosphorylation level of NF-*κ*B was also found at high concentrations of melatonin. No obvious change was observed in JNK activity after melatonin treatment. (d) Melatonin increased the proportion of cells in the S-phase and decreased that of cells in the G0/G1 phase. With U0126, the effects on cell cycle distribution were reduced. (e) Similarly, the expression of the cell cycle-related proteins was reduced in NPCs treated with melatonin and U0126. (f) Quantified analysis of cell cycle-related protein Western blot result. (g, h) The inhibition of apoptosis by melatonin was also reversed upon addition of U0126. (i) Quantified analysis of apoptosis-related protein Western blot result. (j) Western blot analysis also showed that, after addition of U0126, U0126 had a significant weakened role on melatonin in the downregulation of collagen X expression and there is no more positive effect from melatonin on aggrecan and collagen II expression in the NPCs. (k) Quantified analysis of cell matrix protein Western blot result (^∗^*p* < 0.05).

To elucidate the mechanism by which melatonin regulates NPCs, the expression of key proteins in the NF-*κ*B, ERK, and JNK signaling pathways was analyzed by Western blot. Melatonin significantly increased ERK1/2 activity above the concentration of 1 *μ*M. Both the expression of ERK 1/2 and phosphorylated ERK1/2 significantly increased after a treatment of high concentrations of melatonin. A slight inhibition of phosphorylated NF-*κ*B protein expression was detected at 1 *μ*M. A decrease in the phosphorylation level of NF-*κ*B was also found at high concentrations of melatonin, while no obvious change was observed in JNK activity after melatonin treatment (Figures 3(a)–3(c)). Thus, the ERK1/2 pathway was the most sensitive among the three pathways.

To further confirm the interaction between melatonin and ERK signaling pathway, we incubated NPCs with melatonin (0 and 5 *μ*M) to investigate the role of melatonin in NPCs treated with or without an ERK1/2 inhibitor, U0126. Melatonin treatment alone increased the proportion of cells in the S-phase and decreased that of cells in the G0/G1 phase. In the presence of U0126, the effects of melatonin on cell cycle distribution (G0/G1 and S phases) were reduced (Figure 3(d)). Similarly, melatonin alone reduced the expression of p21 and p27 and increased CDK2 and CDK4 levels in NPCs. Compared with melatonin-treated cells, the expression of CDK2 and CDK4 was obviously reduced, while the expression of p21 and p27 was significantly increased in NPCs treated with melatonin and U0126 (Figure 3(f), *p* < 0.05), while the expression of the cell cycle-related proteins of the melatonin and U0126 group has no significant differences compared with that of the control group (Figures 3(e) and 3(f), *p* < 0.05). After the treatment of U0126, the effects of melatonin on cell cycle protein expression are significantly weakened. Consistent with this phenomenon, flow cytometric analysis showed that there were no significant changes in terms of the apoptotic NPC percentages between the two groups after treatment with U0126 (*p* > 0.05), although the percentage in the melatonin group was significantly lower than that in the control group without an ERK1/2 inhibitor (*p* < 0.05) (Figure 3(g)). In addition, melatonin treatment inhibited the apoptosis of NPCs in the absence of U0126 by reducing the expression of Bax and cleaved caspase-3 and increasing Bcl-2. However, the inhibition of apoptosis by melatonin was reversed upon addition of U0126 (Figures 3(h) and 3(i), *p* < 0.05). Western blot analysis for the NPC matrix also showed that melatonin treatment increased the expression of collagen II and aggrecan and decreased that of collagen X levels. After the addition of U0126, U0126 had a significant weakened role on melatonin in the downregulation of collagen X expression, and there is no more positive effect from melatonin on aggrecan and collagen II expression in the NPCs (Figures 3(j) and 3(k), *p* < 0.05).

### 3.6. Melatonin Protects NPCs from Degeneration via the ERK Pathway in a Rabbit IDD Model

**Figure 4:**
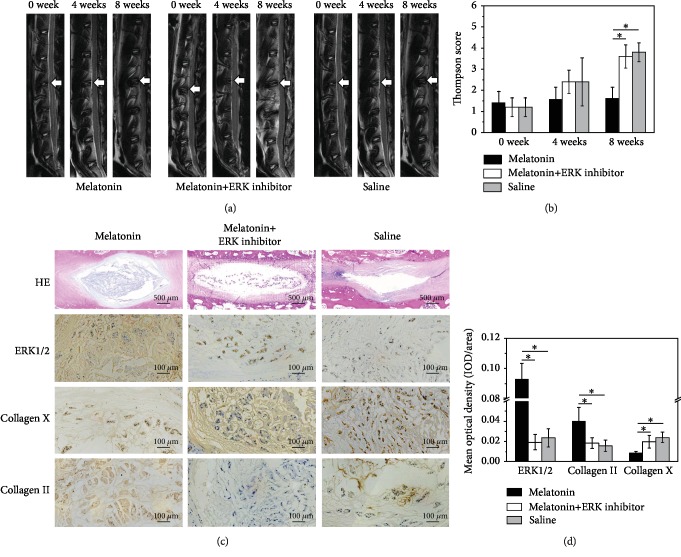
(a) Magnetic resonance imaging findings after melatonin injection on rabbit intervertebral discs. T2 signal intensity was stronger for the melatonin-injected discs than for the saline discs, including the melatonin plus U01256 group. (b) Thompson score showed the significant radiological improvement on the 8th week brought by melatonin (^∗^*p* < 0.05). (c) Histological analysis on intervertebral discs by HE staining. More chondrocytes with intact annulus fibrosus were observed in the melatonin-treated rats. However, reduced number of chondrocytes and twisted or damaged annulus fibrosus presented in the two control groups. ERK 1/2 immunohistochemical staining indicates that ERK 1/2 expression was more apparent in the melatonin group. Quantification of immunohistochemical staining showed that the melatonin group had significantly higher mean optical density than the inhibitor-treated and control groups. Collagen II and X immunohistochemical staining indicates that collagen II was detected in the melatonin-treated group, but little in the two control groups. By contrast, the staining for collagen X was weaker in the melatonin-treated group. Quantification of collagen II immunohistochemical staining showed that the melatonin-treated discs had significantly higher mean optical density than the control groups and the expression of collagen X was significantly lower. (d) Quantified analysis of immunohistochemical staining showed the significant histological improvement brought by melatonin via the ERK pathway (^∗^*p* < 0.05).

Furthermore, a rabbit lumbar intervertebral disc degeneration model was established to investigate the role of melatonin in intervertebral disc degeneration *in vivo*. All rabbits were subjected to T2WI sagittal scanning, and results showed that the intervertebral disc signals were obviously alleviated in the melatonin group compared with the rabbit in the degeneration group and the melatonin plus U0126 group (Figure 4(a)). Though the mean Thompson score of the melatonin group was lower than that of the saline group and the melatonin plus U0126 group, there was no obvious difference in the first 4 weeks after the injection. The Thompson scores of the melatonin group were significantly different from those of the saline group and the melatonin plus U0126 group (*p* < 0.05) in the eighth week (Figure 4(b)). In the saline group and the melatonin plus U0126 group, HE staining presented with shrunken nucleus pulposus in the intervertebral discs, reduced number of chondrocytes, and twisted or damaged annulus fibrosus. However, more chondrocytes with intact annulus fibrosus were observed in the melatonin-treated rabbits (Figure 4(c)). The intervertebral discs were also stained for the major proteins ERK1/2, collagen II, and collagen X to determine the function of the intervertebral discs. Injection of melatonin resulted in an increased expression of collagen II in the intervertebral discs, which was of higher intensity, in comparison to that of the melatonin plus U0126 and saline groups. By contrast, the staining for collagen X was weaker in the melatonin-treated group when compared with the melatonin plus U0126 and saline groups (Figures 4(c) and 4(d), *p* < 0.05). Compared with the two control groups, the melatonin group showed a significant increase in ERK1/2 according to immunohistochemical staining. This suggested that melatonin could activate the biological activity of ERK1/2, which effectively promotes the synthesis and secretion of main matrix components that contribute to the structural and functional recovery of intervertebral disc degeneration.

## 4. Discussion

The etiology of IDD is various, among which apoptosis is one of the most important. In 2001, for the first time, Ariga et al. revealed the positive correlation between apoptosis and IDD [23]. Many studies also indicated that apoptosis initiated the process of IDD [24–26].

Our study investigated the effect of melatonin on NPC behaviors and its underlying mechanism. Cell viability is an important factor for cell survival, which is also an important biological characteristic of IDD. We performed CCK-8 assay to detect NPC viability. Results clearly indicated that treatment with melatonin at different concentrations increased the viability of cells and retarded the morphological changes in the degenerated NPCs. The cell cycle is governed positively by cyclin/CDK complexes and negatively by CDKIs. The cyclin D/CDK4 and cyclin E/CDK2 complexes are required for cell cycle progression from the G1 phase to the S phase [27]. CDKIs, such as p21 and p27, inhibit cyclin/CDK activity, resulting in cell cycle arrest in the G0/G1 phase. Indeed, our results showed a dose-dependent increase in the percentage of cells in the S phase and a decrease of the percentage of NPCs in the G0/G1 phase after melatonin treatment by downregulation of CDK2 and CDK4 and upregulation of p21 and p27. The intrinsic apoptosis pathway is one of the two classic apoptosis pathways. In this pathway, caspase-3 plays an important role and can be activated by upstream effector proteins and induce the apoptosis cascade [28]. The Bcl-2 family, including antiapoptotic protein Bcl-2 and proapoptotic protein Bax, is the central regulator in the mitochondrial apoptosis pathway [29]. In the present study, melatonin induced the expression levels of Bcl-2 and reduced the expression of Bax in the NPCs, suggesting that melatonin induced antiapoptosis via the apoptotic caspase pathway.

As is well-known, apoptosis is regulated by a variety of intracellular signal transduction cascades. Mechanically, we examined the changes of three common apoptosis-related signaling pathways, NF-*κ*B, ERK, and JNK signaling pathways. Results showed that melatonin obviously increased ERK1/2 activity, slightly inhibited NF-*κ*B activity, and had no effect on JNK expression. The mitogen-activated protein kinase (MAPK) signaling pathway is one of the most important signal transduction pathways and is found widely in cells [30]. Numerous studies have shown [31–34] that extracellular signaling-regulated kinase (ERK) pathway signal pathway, one of the subtypes of MAPK pathway, is a major participant in the regulation of cell growth, differentiation, and other functions. It participates in the extracellular signal transmission into the nucleus; through nuclear translocation, it transmits mitotic signals and activates transcription factors to promote cell proliferation and inhibit apoptosis [35]. The ERK pathway can be activated by inflammatory factors [36, 37] such as TNF-*α* and IL-1. The activation factors activate tyrosine kinase via cell membrane receptor. The signal was then passed to Ras and then activate Raf, MEK1/2 (MAPK/ERK kinase), and ERK1/2 sequentially [31, 38], regulating the transcription and expression of protein. Many studies have shown that the ERK pathway is involved in cell cycle regulation, especially the G1/S phase [39–41].

Recently, many studies have shown that the ERK signaling pathway plays an important role in IDD [6–9]. In 2005, Risbud et al. [42] found that rat NPCs could downregulate apoptosis by reducing the expression of apoptosis genes through the activation of ERK pathways. Pratsinis et al. [43] revealed that the proliferation of intervertebral disc cells can be enhanced by PDGF, bFGF, and IGF-I via ERK signaling pathways. Several studies have further proved that the ERK pathway is a potential target for the treatment of IDD and the activated ERK signal pathway can reverse or delay the process of IDD, which is consistent with the current study. Results showed that melatonin obviously increased ERK1/2 activity [44–47]. Given that melatonin elevated the expression of ERK1/2 in the NPCs, we thus speculated that melatonin could possibly activate the ERK1/2 signaling pathway to affect the function of NPCs. To confirm this hypothesis, the ERK1/2 pathway was blocked by the addition of an ERK1/2 inhibitor, U0126. Our results showed that the inhibition of ERK1/2 signals reversed the effect of melatonin on the activation of the cell cycle and apoptosis. Western blot analysis showed that the addition of the ERK inhibitor reduced the expression of collagen II and aggrecan, which were upregulated by melatonin and increased the expression of type X collagen, suggesting that U0126 aggravated the degeneration of NPCs. Therefore, our data suggest that the ERK1/2 signaling pathway mediates the effect of melatonin in the regulation of the biological processes of NPCs.

A recent work reported by Chen et al. [21] has well demonstrated the positive effect of melatonin on rat caudal tail IDD. However, the test and description for rat intervertebral disc are only about morphology. In addition, the rat tail disc degeneration model, which Chen used, has a quite different geometry and structure from human disc [48]. So, the mechanism underlying the effect of melatonin in the regulation of intervertebral discs was also confirmed *in vivo* by using a rabbit lumbar model of intervertebral disc degeneration, which has a more similar structure to human disc [48] and was induced by needle puncture. There are notochordal cells in the rabbit intervertebral disc, which is different from human adult NP cells. As a result, possible species-related differences in the response to melatonin should be considered. In the current study, we tried our best to avoid the effect of notochordal cells. On the one hand, rabbits have large numbers of notochordal cells at least until 12 months of age [49]. So one-year-old New Zealand white rabbits were selected for the study. On the other hand, the histology of the disc after puncture showed changes in the cell population from notochordal cells to chondrocytic cells [50]. So the injection was performed 4 weeks after the puncture. Four weeks after the injection, MRI examination revealed the delaying effect of melatonin in IDD. Melatonin injection retarded the degeneration of the intervertebral discs, which was finally confirmed by MRI and histological examination. However, combined treatment with an ERK1/2 signaling pathway inhibitor, U0126, reversed the protective role of melatonin in IDD.

Melatonin is a well-known neuroendocrine hormone secreted by the pineal body, which is implicated in the regulation of circadian and seasonal rhythms. Moreover, melatonin exhibits antitumor, antiosteoporosis, anti-inflammation, and antioxidation properties. In addition, melatonin has been used for hypnosis, anticancer therapy, anticardiovascular and cerebrovascular diseases, and antiaging [51–55]. In a preclinical study, melatonin was applied for osteoarthritis treatment for its ability to repair cartilage defects and promote cartilage regeneration. Moreover, melatonin presents a low toxicity and is a cheap agent with a wide range of applications. Melatonin has a synergistic effect when combined with other agents. Our study could provide the theoretical and experimental basis for the clinical application of melatonin by revealing the mechanism underlying its protective property against IDD for the very first time. This discovery opens up a new therapeutic approach for clinical treatment of IDD.

In summary, we conclude that melatonin affects the intervertebral discs in multiple ways. Melatonin increases the NPC viability through inhibition of cell cycle arrest and apoptosis, increases the secretion of functional factors to influence NPC physiological function, and finally retards cell degeneration. The current study provides the theoretical and experimental basis for developing a melatonin-based therapy for IDD via the ERK 1/2 signaling pathway.

## Data Availability

The data that support the findings of this study are available from the corresponding author upon reasonable request.
